# Supplementary material for: Study on Characteristics and Lignification Mechanism of Postharvest Banana Fruit during Chilling Injury
Source: Foods. 2023 Mar 4;12(5):1097. doi: 10.3390/foods12051097 (PMC10000439; doi:10.3390/foods12051097)
Supplement: Supplementary file 1 [file foods-12-01097-s001.zip › foods-2131199-supplementary.pdf]

Supplementary material

Figure S1. Changes of amylase activity in banana peel during storage at 6°C and 25°C. α-amylase activity (A), β-amylase activity (B). Each data point represents a mean ± standard error (n=3). '*' means a significant difference between control and experimental fruit at 5 % level.

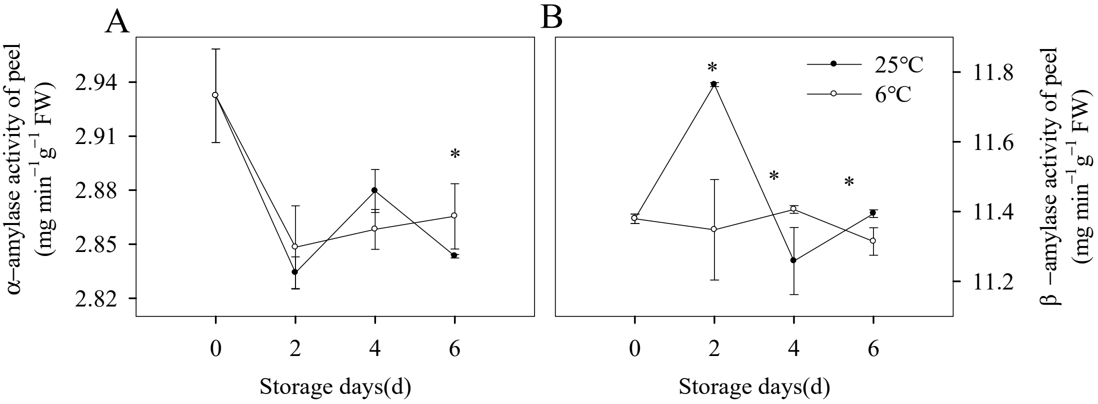

Figure S2. Ultrastructural changes of banana peel during storage at 6°C and 25°C were observed by scanning electron microscope. Parenchyma cells (A) and vascular cells (B) of banana peel during storage.

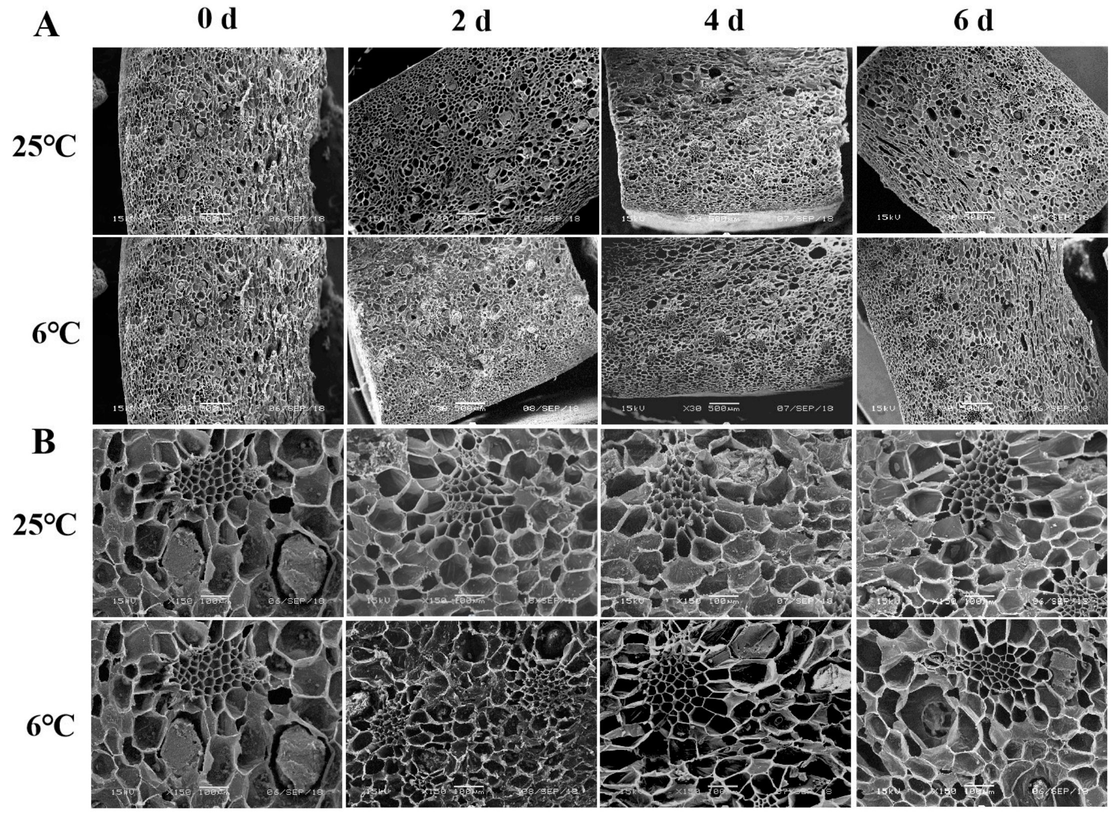

Table S1. Primers used for RT-qPCR.

Gene Name	Gene ID	Forward（5'-3'）	Reverse（5'-3'）
POD1	Ma02_t04930.1	ACAACAACGTGGCTGAGAAGGA	GCAGTCGGTGGTGGCAGATTA
PAL	Ma01_t04420.1	CACAGGAGGACCAAGCAAGGAG	GCAGCGGACGAAGGCAATGT
LAC3	Ma01_t22390.1	ACATCTCCGACGCATTCACCAT	CCGAGCATGAGCACCGAAGT
CCR4	Ma01_t00690.1	AGGAGGAAGGTGTGGACGTGAAG	GGCAACGGCTTACAAGTGAGGAT
CAD2	Ma04_t12960.1	GCAGCCTCAAGACGGAGAAGAC	GGTGGAGATCAGTGTGGCAGAC
4CL7	Ma09_t07500.1	ACGATCACCAACGGCAACACC	CCTTCAACCTGTCCAGCACGAA
MaAtion-3	Ma09_t08490.1	TGGTATGGAAGCCGCTGGTA	TCTGCTGGAATGTGCTGAGG
